# The role of the purinergic P2X_7 _receptor in inflammation

**DOI:** 10.1186/1476-9255-4-5

**Published:** 2007-03-16

**Authors:** Martin F Lister, John Sharkey, Deborah A Sawatzky, Joseph P Hodgkiss, Donald J Davidson, Adriano G Rossi, Keith Finlayson

**Affiliations:** 1 MRC Centre for Inflammation Research, The Queen's Medical Research Institute, The University of Edinburgh, 47 Little France Crescent, Edinburgh, EH16 4TJ, UK; 2 Astellas CNS Research in Edinburgh, The Chancellor's Building, The University of Edinburgh, 49 Little France Crescent, EH16 4SB, UK

## Abstract

The inflammatory process, orchestrated against a variety of injurious stimuli, is composed of three inter-related phases; initiation, propagation and resolution. Understanding the interplay between these three phases and harnessing the beneficial properties of inflammation whilst preventing its damaging effects, will undoubtedly lead to the advent of much needed therapies, particularly in chronic disease states. The P2X_7 _receptor (P2X_7_R) is increasingly recognised as an important cell surface regulator of several key inflammatory molecules including IL-1β, IL-18, TNF-α and IL-6. Moreover, as P2X_7_R-dependent cytokine production is driven by activating the inflammasome, antagonists of this receptor are likely to have therapeutic potential as novel anti-inflammatory therapies. The function of the P2X_7_R in inflammation, immunity and its potential role in disease will be reviewed and discussed.

## 1. Background

Inflammation is an important physiological reaction which occurs in response to a wide variety of injurious agents (e.g. bacterial infection or physical trauma) ultimately aiming to perform the dual function of limiting damage and promoting tissue repair [1]. The inflammatory process is often viewed as being comprised of three closely linked phases: – initiation, propagation and resolution, with current anti-inflammatory therapies designed to limit or prevent the initiation and propagation phases. However, it is increasingly recognised that therapies aimed at enhancing the resolution phase will be important in limiting the damage associated with persistent inflammatory disease states such as rheumatoid arthritis, chronic obstructive pulmonary diseases and artherosclerosis [2].

In recent years, the role of ATP and its cognate receptors in the inflammatory process has been recognised. In particular, the P2X_7 _receptor (P2X_7_R) which is expressed primarily (though not exclusively) on cells of haemopoietic origin [3] is thought to play an important role in macrophage/microglial and granulocyte function by regulating cytokine production and apoptosis. Moreover, as the P2X_7_R is known to be up-regulated during inflammation, antagonists of this receptor may serve as novel anti-inflammatory agents. In this review we summarise recent advances in the understanding of the role of the P2X_7_R in inflammatory processes and highlight the potential of P2X_7_R ligands for the treatment of chronic inflammatory diseases, focusing particularly on tuberculosis and cancer.

## 2. P2X_7 _Receptor Pharmacology

Extracellular ATP is known to activate two classes of membrane-bound receptors; the metabotropic P2Y (P2Y_1_, P2Y_2_, P2Y_4_, P2Y_6 _and P2Y_11–14_), and ionotropic P2X (P2X_1–7_) receptors with the pharmacology, distribution and putative functions of these receptors extensively reviewed [4-6]. Of the P2 receptors, the P2X_7_R has attracted considerable interest as a consequence of its unique biological properties. Brief activation of the P2X_7_R by ATP or its stable analogue 2',3'-O-(benzoyl-4-benzoyl)-ATP (BzATP) results in the opening of a non-selective cationic channel. However, upon prolonged stimulation, the P2X_7_R forms an aqueous pore that allows the passage of hydrophilic molecules of up to 900 Da, which can ultimately lead to cell death [7], probably by colloido-osmotic lysis [8]. In contrast, transient receptor activation can induce pseudoapoptosis, a process which is readily reversible [9]. The activation of this receptor has now been associated with the stimulation of a plethora of downstream signalling cascades resulting in the release of a number of inflammatory mediators. Principle amongst these is interleukin-1β (IL-1β), the processing and release of which is critically dependent upon P2X_7_R activation and is discussed extensively below. As with all P2X receptors, elucidating the role of the P2X_7_R has been hampered by a paucity of receptor selective agonists and antagonists. BzATP, widely viewed as a selective agonist of the P2X_7_R, exhibits greater potency for other P2X and P2Y receptors [10-12]. Similarly, it is important to appreciate that oxidised ATP (oATP), although often presented as a P2X_7_R-specific antagonist, can attenuate pro-inflammatory signalling by mechanisms distinct from P2X_7_R activation [13,14]. Although a number of putatively selective P2X_7_R antagonists have recently been described [15-17], the effects of these agents in animal models of disease has yet to be published.

## 3. The role of the P2X_7_R in inflammatory cell function

Since nucleotides (such as ATP) are normally retained within the cytoplasm of a cell, their presence in the external milieu (e.g. during the process of cytolysis [7]) are thought to provide 'danger' signals, inducing antigen presenting cells to initiate the innate immune response [18]. Importantly, innate immunity can be initiated by a variety of cytokines such as IL-1β, IL-18, IL-6 and tumour necrosis factor-α (TNF-α), all of which can be produced by P2X_7_R activation (*vide infra*). In contrast, chronic exposure to low-dose ATP activates dendritic cells and macrophages to secrete anti-inflammatory cytokines (IL-10 and IL-1 receptor antagonist (IL-1RA)) suppressing inflammation and favouring the development of a Th2 response [18]. These observations suggest that the immune and/or inflammatory response can be redirected when deemed to be detrimental to the host. The putative role of the P2X_7_R in such processes is discussed below.

### 3.1. P2X_7_R regulation of cytokine production in haemopoietic cells

It has been clear since the cloning of the P2X_7_R 10 years ago [19], that this channel is predominantly expressed on cells of haemopoietic origin such as monocytes, macrophages and microglia. More importantly, as activation of these cell types is associated with increased expression of the P2X_7_R, this ultimately leads to an amplification of the downstream production of the pro-inflammatory cytokines IL-1β and IL-18, and in turn IL-6, IL-8 and TNF-α. As over-production of these cytokines is detrimental, particularly in chronic disease states, and underlies the pathophysiology of a range of peripheral and central disorders, controlling their release is paramount.

#### 3.1.1. The role of P2X_7_R in IL-1β production

In recent years, a great deal of attention has been devoted to elucidating the mechanisms of release of the pro-inflammatory leaderless cytokine IL-1 from monocytes and macrophages. Originally produced as 31-kDa precursors, the two IL-1 isoforms, pro-IL-1α and pro-IL-1β, are subsequently cleaved by interleukin-converting enzyme (ICE; also known as caspase-1 [20]) to produce the mature 17-kDa forms [21]. IL-1α and IL-1β are thought to have identical biological actions, although IL-1β, unlike IL-1α, is inactive in its immature form [21]. The mechanism of IL-1β release has been extensively studied *in vitro*, although there are only a limited number of molecules capable of inducing controlled release, and whether these processes reflect the *in vivo *situation remains unclear. Upon release, IL-1β is known to elicit diverse responses, including the activation of macrophages, T-cells and signalling cascades, as well as the induction of cyclooxygenase type 2 (COX-2) and fever [22]. IL-1 has been shown to be important in many diseases including rheumatoid arthritis [23], multiple sclerosis [24], asthma [25] and chronic obstructive pulmonary disease [26]. It is therefore clear that IL-1β is of particular importance in the initiation and propagation of an inflammatory response, with its functions and therapeutic potential extensively reviewed [22,27].

**Figure 1:**
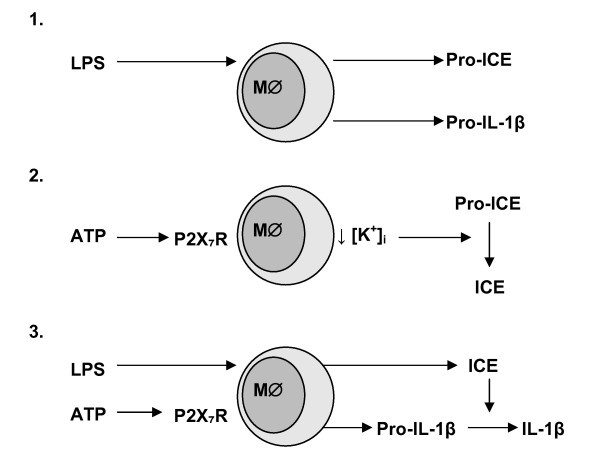
**Summary of the production of active IL-1β**. This process can be divided into 3 stages. Stage 1: LPS stimulates monocytes/macrophages (M∅) to produce pro-ICE and pro-IL-1β. Stage 2: ATP stimulates the P2X_7_R expressed on M∅ to cause a fall in intracellular K^+ ^concentration ([K^+^]_i_) which in turn converts pro-ICE to ICE. Stage 3: LPS-primed M∅ following ATP stimulation results in activated ICE which converts inactive pro-IL-β to active IL-1β. It should be noted that this process is intracellular and the figure is for illustrative purposes only (see text for references).

Originally, cell death by apoptosis was reported to stimulate the production and release of mature IL-1β, although the mechanism was not identified [28]. The release of mature IL-1β appeared to require two consecutive stimuli [29], with LPS stimulation in monocytes only producing pro-ICE and pro-IL-1β [30]. The latter authors reported that ATP-stimulated K^+ ^efflux was important for the release of mature IL-1β [30], with Ferrari and colleagues subsequently suggesting that it was P2X_7_R-mediated, and independent of apoptosis [31]. This was latterly confirmed in pharmacological [32] studies and those using P2X_7_R knockout mice [33,34], with the activation of the P2X_7_R by ATP producing a fall in cytoplasmic K^+ ^concentration which in turn stimulates processing of pro-ICE to ICE, and thereby inducing release of mature IL-1β (Figure 1; [35]). Indeed, in an elegant series of studies Surprenant and colleagues have subsequently demonstrated that ATP-induced activation of the P2X_7_R results in the shedding of microvesicles which contain mature IL-1β [36] and more recently IL-1RA [37]. With high concentrations (0.5–5 mM) of ATP required for optimal activation of P2X_7_R-mediated IL-1β release *in vitro *[38], alternative endogenous agonists that could produce significant P2X_7_R stimulation have been sought. Interestingly, several cationic host defence peptides (CHDP; also known as antimicrobial peptides) have recently been shown to mediate post-translational processing of IL-1β in LPS-primed monocytes. Although the mechanisms of action of the porcine CHDP protegrin-1 and -3 have been shown to be P2X_7_R-independent [39], three studies have now proposed that P2X_7_R activation underlies some of the immunomodulatory effects of the human CHDP, LL-37 [38,40,41]. LL-37 is the major active cleavage product of the only human cathelicidin hCAP18, is upregulated in infection and inflammation [42,43], and in addition to broad-spectrum antimicrobial activity and direct anti-endotoxic effects, LL-37 has a number of immunomodulatory roles [44]. LL-37 has now been shown to induce caspase-1 activation and secretion of mature IL-1β in LPS-primed monocytes, in the absence of cytotoxicity, through P2X_7_R activation [38]. Furthermore, recent studies have demonstrated that concentrations of LL-37 as low as 250 ng/ml, and well within the physiological range, can inhibit apoptosis in human neutrophils, in a P2X_7_R-dependent manner involving the PI3-kinase pathway [40,41]. Such studies indicate that in addition to extracellular ATP, the endogenous, inducible CHDP, LL-37 may activate the P2X_7_R on key innate immune effector cells to modulate cytokine release. Finally, as compounds such as Tenidap, which is being evaluated for its anti-inflammatory and anti-arthritic properties also appear to inhibit the release of IL-1β [45], whilst sensitising the P2X_7_R on macrophages to the cytotoxic effects of ATP [46], future studies may show that the P2X_7_R could be regulated by a range of ligands.

The importance of, and the mechanisms through which the P2X_7_R regulates the production of the pro-inflammatory cytokines IL-1β and IL-18, and potentially the innate immune response, was recently and beautifully described by Mariathasan and colleagues [47]. These authors showed that the P2X_7_R is up-stream of the inflammasome, an important complex of cytosolic proteins that are known to regulate caspase-1 activation and ultimately the processing of IL-1β and IL-18. With inflammasome dysregulation known to produce inflammatory disorders such as Muckle-Wells syndrome and neonatal onset multisystem inflammatory disease, it is clear that inhibiting inflammasome activation with P2X_7_R antagonists could affect the outcome of a range of inflammatory disorders [47]. However, one must remember that the P2X_7_R may not be the only purinergic receptor involved in IL-1β release. A recent study has shown ATP-dependent Ca^2+ ^release from intracellular stores (endoplasmic reticulum) is also involved in the secretion of pro-IL-1β, although it was not independently capable of releasing mature IL-1β [48]. As discussed above, K^+ ^efflux was also reported to be necessary for the release of mature IL-1β, with Brough and colleagues (2003) proposing that ATP may stimulate both P2X and P2Y receptors [48]. The importance of P2Y receptor stimulation and Ca^2+ ^release from intracellular stores remains to be determined.

P2X_7_R-mediated regulation of IL-1β has also been demonstrated within the central nervous system where microglia are the resident monocytic cells. In a seminal study in 1997, Ferrari *et al *[49] reported that ATP induced IL-1β production in cultured microglial cells through the activation of the P2X_7_R. Subsequent studies showing that cultured microglia from P2X_7_R knockout mice do not release IL-1β following exposure to LPS and ATP [50] support the role for P2X_7_R in IL-1β production, albeit *in vitro*. P2X_7_R up-regulation has been observed in response to a variety of inflammatory brain insults, underpinning the view that P2X_7_R antagonists may be of therapeutic use for the treatment of several disorders including stroke, traumatic brain injury (TBI), multiple sclerosis and Alzheimer's disease [3,51-53]. Since IL-1β has been reported to induce COX-2 in various tissues including glia, it has been proposed that a vicious cycle occurs whereby ATP release (from cell death for example) leads to P2X_7_R activation, IL-1β release, COX-2 induction and further cell death with consequent ATP release; this type of self-perpetuating cycle may underlie lesion expansion particularly in stroke and TBI. Once selective P2X_7_R antagonists become commercially available it will be possible to test the importance of this receptor in these processes. However, it is interesting to note that non-specific antagonism of P2X receptors by PPADS, and the inhibition of IL-1β, and COX-2, have all been reported to be effective in animal models of stroke and other neurodegenerative disorders [51,54]. Intriguingly, another function attributed to the P2X_7_R that is important in neuropathology is microglial production of superoxide anion [55]. The significance of P2X_7_R regulation of superoxides was underlined by the observation that P2X_7_R expression was up-regulated around β-amyloid plaques in a mouse model of Alzheimer's disease [55]. It was also subsequently shown that in human microglia, β-amyloid-induced cytokine release (e.g. IL-1β) was found to be modulated by ATP, probably via the P2X_7_R [56].

Understandably, polymorphisms in the genes encoding IL-1, its receptor, and IL-1RA have been found to be associated with a range of diseases including rheumatoid arthritis, systemic lupus erythematosus, atherosclerosis and tuberculosis [57]. As a result of the importance of the P2X_7_R in IL-1β processing and release, polymorphisms in this unique ion channel have been investigated and to date, in excess of 260 polymorphisms have been identified for the P2X_7_R [58,59]. One such polymorphism is the single nucleotide substitution at position 1513 of the P2X_7_R gene which changes a glutamic acid to an alanine at amino acid position 496 (Glu^496^Ala), and leads to loss of function of the receptor [60]. It is interesting to note that this polymorphism decreased the ATP-induced K^+ ^efflux subsequently delaying the ATP-induced release of IL-1β. The fact that IL-1β release was delayed rather than abrogated indicates that there are compensatory or redundant mechanisms present [61]. However there is now evidence from P2X_7_R polymorphism studies, that those associated with a loss of function mutation have a reduced sensitivity to inflammation [62].

In the absence of commercially available potent and selective P2X_7_R antagonists, P2X_7_R knockout mice have provided new insights into the *in vivo *role of this receptor. Labasi and colleagues [34] reported that peritoneal macrophages from P2X_7_R deficient mice were unable to produce mature IL-1β in response to LPS, or ATP application, or with a combination of both stimuli. This study also compared the induction of monoclonal anti-collagen-induced arthritis in P2X_7_R-deficient mice and wild-type littermates, with the former group demonstrating reduced susceptibility to, and severity of disease [34]. It was therefore suggested that, in normal mice, endogenous ATP is present in sufficient concentrations at sites of inflammation to activate the P2X_7_R [34], (an area that has attracted some scepticism based on *in vitro *work with the addition of exogenous ATP [38]). However, as described earlier, care must now be taken in interpreting results observed *in vivo*, as although ATP was originally thought to be the only endogenous agonist of the P2X_7_R, recently other physiological agents such as LL37 (see above) and NAD [63] have been reported to activate the P2X_7_R at lower concentrations. New studies in P2X_7_R knockout mice continue to indicate that this receptor plays a role in a number of conditions in addition to arthritis and include multiple sclerosis, hepatitis and pain [34,64-66].

#### 3.1.2. The role of P2X_7_R in IL-18 production

In addition to IL-1β secretion, the P2X_7_R has been implicated in the synthesis and release of the related leaderless cytokine IL-18 (interferon-γ-inducing factor), which is also produced through cleavage of pro-IL-18 by ICE [47,59,67], although it has not yet been extensively studied. In contrast to IL-1β, secretion of IL-18 was found to be less dependent on LPS-priming [68], although conflicting data was presented by Mehta *et al *who found IL-18 production to be LPS-dependent [69]. Indeed it has been shown that individuals expressing the Glu^496^Ala P2X_7_R polymorphism produce significantly less IL-18 when their monocytes are stimulated by ATP [61]. We have also shown that in LPS primed, BzATP stimulated, human monocytic THP-1 cells, both IL-1β and IL-18 release is inhibited by P2X_7_R antagonists (Finlayson *et al*., unpublished observations). The importance of IL-18 in general inflammatory processes, and its suitability as a therapeutic target have been extensively discussed [70], however the simultaneous inhibition of both IL-1β and IL-18 by P2X_7_R antagonism has its obvious attractions.

#### 3.1.3. The role of P2X_7_R in TNF-α production

In general, TNF-α is regarded as a pro-inflammatory cytokine that is produced in response to injury, exerting a number of important roles in the immune system and during inflammatory responses. It is of particular interest in neuropathology where this dual role is most clear, with TNF-α having both neurotoxic and neuroprotective effects [71-74]. It appears that microglia, the principal immune cells of the central nervous system, have enhanced P2X_7_R expression following inflammatory insults (see above) [3,75]. However, as mentioned previously, ATP may act as a 'danger' signal, which recruits microglia to damaged areas of the brain through P2Y rather than P2X receptors [76]. In a rat model of neuronal injury, stimulation of the P2X_7_R by ATP has been shown to protect neurones by releasing TNF-α [77]. In contrast to TNF-α release in rat microglia, Kucher and Neary reported that the P2X_7_R was probably responsible for the inhibition of TNF-α release in rat LPS-stimulated astrocytes [78]. Indeed, these authors proposed that this could be a mechanism to sense the severity of damage and alter the inflammatory response appropriately. There are also some reports by Perregaux *et al *[68] that show ATP alters TNF-α production in human monocytes. As the effects of TNF-α in the CNS will be dependent upon the circumstances of its release, and may differ during the acute response to injury versus the long-term recovery from injury [79], it is vital to understand these effects to facilitate the development of novel therapeutic agents.

In addition to the effects that P2X_7_R polymorphisms have on IL-1β production, it has also been noted that individuals harbouring such polymorphisms have reduced plasma TNF-α levels (but higher levels of the anti-inflammatory cytokine IL-10) relative to normal subjects [62]. Results from this study suggested that during infectious perturbations, 15% of healthy individuals exhibited anti-inflammatory mediator responses, which was correlated with the level of P2X_7_R pore activity. While normal pore activity appeared to increase microbial clearance, reduced pore activity may provide some protection from autoimmune disorders as those with an anti-inflammatory cytokine profile are less likely to mount an adaptive immune response to self tissues [62]). Since the P2X_7_R is important in the production of both TNF-α and IL-1β and as inhibitors of both are in clinical use for the treatment of rheumatoid arthritis [80] and other inflammatory conditions, such observations possibly underlie why AstraZeneca, Pfizer and Abbot amongst others are currently developing P2X_7_R antagonists.

#### 3.1.4. The role of P2X_7_R in IL-6 Production

In rheumatoid arthritis ATP is found in the synovial fluid where a number of P2X_7_R-expressing cells including macrophages are present [81,82]. In joint diseases such as rheumatoid arthritis and in other conditions such as atherosclerosis the P2X_7_R has also been implicated in the secretion of the pro-inflammatory cytokine IL-6 from fibroblasts [83]. In atherosclerosis fibroblasts are likely to be exposed to increased concentrations of ATP because of its secretion from platelets and at sites of chronic inflammation [84]. In a more recent study the same authors have shown that fibroblasts from type-2 diabetic patients have increased sensitivity to ATP, which is likely to contribute to diabetic vascular disease [85]. Furthermore, although mast cells have received little attention with regard to the P2X_7_R, it has been known for some time that these cells express this unique receptor (originally described as the P2Z receptor) along with several other P2X and P2Y receptors [86]. In addition to inducing cell death, ATP-stimulation of the P2X_7_R on murine mast cells has been shown to increase the expression of several pro-inflammatory cytokines, including IL-6 and TNF-α [87]. Considering the role of mast cells, especially in allergic inflammation, it would appear pertinent to re-examine the role of the P2X_7_R given its therapeutic potential in this area. Finally, new *in vivo *evidence has been presented supporting the use of P2X_7_R antagonists as anti-inflammatory and antipyretic agents (where excessive pro-inflammatory cytokine production or high fever is harmful to the host [88]). These authors provided important new insights into LPS-induced febrile response in rats, and showed that the ATP released from activated immune cells stimulated cytokine release which then initiated the febrile response [88]. These authors suggested that the P2X_7_R plays a central role [88], which is perhaps unsurprising given that the cytokines IL-6, IL-1β and TNF-α all act as endogenous pyrogens [89].

### 3.2. P2X_7_R regulation of granulocyte function and cell death

It is well known that granulocytes play a critical role in acute inflammation, with polymorphonuclear neutrophils (PMNs; 95% of circulating granulocytes) and eosinophils of particular interest. PMNs are phagocytic cells that play a critical role in the host defence against bacterial and fungal infections, whereas eosinophils are primarily involved in the host defence against parasites, and function in the pathogenesis of allergic and immunological disease. In general, granulocytes are recruited to sites of inflammation where they release inflammatory mediators such as leukotriene B_4_, platelet activating factor and IL-8. However in the event of the failed clearance of apoptotic PMNs these inflammatory mediators can lead to tissue destruction and are thought to underlie the pathophysiology of diseases such as asthma, rheumatoid arthritis and atopic dermatitis [90-92].

#### 3.2.1. P2X_7_R mediated modulation of apoptosis in PMNs

**Figure 2:**
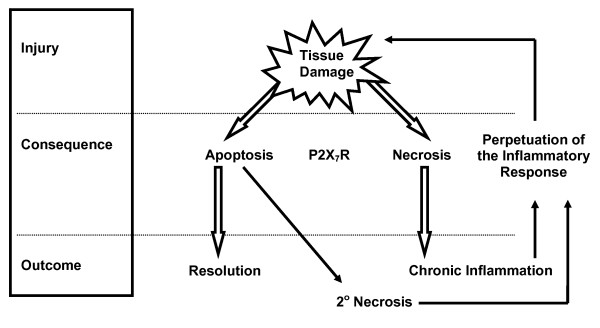
**Possible outcomes of an inflammatory response**. Tissue damage (inflammation initiation) can lead to cell death by apoptosis or necrosis. The balance between these two types of cell death can determine the outcome of the inflammatory response e.g. propagation (leading to chronic inflammation) or resolution. Resolution is more common when cell death is predominantly apoptotic, however, the phagocytosis of apoptotic or necrotic cells is also an important determinant of the outcome of inflammation. As can be seen, the P2X_7_R may be critical to determining the outcome of an inflammatory response.

The process of cell death is fundamental to many aspects of physiology and pathophysiology, and of great importance to the regulation of inflammation. Apoptosis is a process of controlled cell death in which cells undergo well characterised morphological changes, including the classical features of chromatin condensation, cell shrinkage, and the formation of apoptotic bodies [93]. In contrast to necrotic cell death, apoptotic cell death is a predominantly non-inflammatory process in which the membranes of cells remain intact. This allows the cytotoxic granule contents of cells such as PMN to remain enclosed within the cytoplasmic membrane while the cell is phagocytosed, thereby minimising tissue damage. Furthermore, phagocytosis of apoptotic cells, unlike other particles, has been shown to inhibit the release of pro-inflammatory mediators including IL-1β, IL-8 and TNF-α [94]. However, failure of rapid phagocytosis can result in secondary necrosis of the apoptotic cell leading to tissue damage and inflammatory infiltrate (Figure 2). Thus, regulation of innate immune effector cell apoptosis, in particular that of short-lived granulocytes, is critical to the induction, maintenance and resolution of inflammatory processes [95]. Apoptosis is regulated at a cellular level by the expression and activation of the Bcl-2 family of proteins and the components of the caspase pathways, which dictate the lifespan and mode of cell death in such cells [96]. Importantly, recent studies indicate that P2X_7_R activation may modulate a number of cell death processes through effects upon these key regulators of apoptosis.

As described above, the human cathelicidin LL-37 inhibited PMN apoptosis in a P2X_7_R-dependent manner [40,41]. Stimulation of PMN with LL-37 was shown to upregulate expression of the Bcl-2 family protein Mcl-1, a key rapid response component which promotes PMN survival [97], and to inhibit the cleavage and activation of the critical apoptotic regulator pro-caspase-3 [98,99]. Interestingly, whereas lower levels of LL-37 acted primarily as a neutrophil survival factor, higher levels appeared to promote necrotic cell death while inhibiting apoptosis [41]. Thus stimulation of the P2X_7_R has the capacity to exert a potent effect upon neutrophil survival. These data indicate that PMN express functional P2X_7_R, but the cellular localisation of these receptors in this cell type remains unclear. P2X_7_R expression on human cells has been demonstrated on PMN, HL-60 promyelocytes and granulocytic differentiated cells, and is reported to increase with granulocytic differentiation [100]. However, one report has suggested that human PMN have an intracellular pool of P2X_7_R, with little or no surface expression [101]. Irrespective, these studies suggest that P2X_7_R activation might extend the lifespan of PMN at sites of infection and inflammation, and modulate the mechanism of cell death in these cells.

In contrast to the effects observed in neutrophils, prolonged P2X_7_R activation with extracellular ATP has been shown to induce apoptosis in other cell types, including mast cells and epithelial cells [9,102,103]. In addition, murine whole blood exposed to ATP demonstrated a near complete loss of monocytes, and a decrease in lymphocytes, but no change in PMN numbers [34]. This effect was not seen in P2X_7_R-deficient mice, indicating a P2X_7_R-mediated induction of cell death in these cells [34]. This induction of apoptosis has been proposed to involve the opening of cation-selective membrane pores, and to be a calcium-independent, ROCK-1-dependent pathway [9]. Whereas prolonged or excessive P2X_7_R activation with ATP induces apoptosis, transient activation induces a state of pseudoapoptosis in epithelial cells [9]. Under these conditions, P2X_7_R activation results in a series of very rapid and reversible effects, including calcium-dependent translocation of plasma membrane phosphatidylserine, loss of mitochondrial membrane potential (without cytochrome c release), disruption of the actin filament/microtubule network and membrane blebbing. These data suggest that the P2X_7_R can be associated with two different pathways, inducing pseudoapoptosis or apoptosis in epithelial cells. These effects on cell death, assuming the physiological ligand is ATP, are most likely to occur at sites of tissue damage where ATP is released in considerable quantities [104]. Interestingly, LL-37 has also been shown to induce eukaryotic membrane permeability [38] and been implicated in the induction of apoptosis in epithelial cells [105]. Thus, although the possible role for P2X_7_R in mediating these latter effects remains to be determined, it is tempting to speculate that alternative agonists such as LL-37 could induce P2X_7_R-dependent apoptosis, and the safe removal of infected cells in an inflammatory environment, even in the absence of high concentrations of ATP.

Thus, an intriguing contrast exists between the effects of P2X_7_R stimulation on cell death pathways in different host innate immune effector cells. Nevertheless, the consequences in each case may enhance the inflammatory response and the clearance of infection in acute infection, but have potentially deleterious effects in chronic inflammatory conditions. Indeed, Chen and Brosnan have shown P2X_7_R knockout mice to be more susceptible to autoimmune encephalomyelitis (a model for multiple sclerosis), attributing this susceptibility to reduced apoptotic activity in lymphocytes [64]. A further understanding of these processes is anticipated to facilitate the development of novel therapeutic agents capable of modulating inflammation via P2X_7_R-mediated effects on cell death pathways.

#### 3.2.2. P2X_7_R and cytokine production in eosinophils

**Figure 3:**
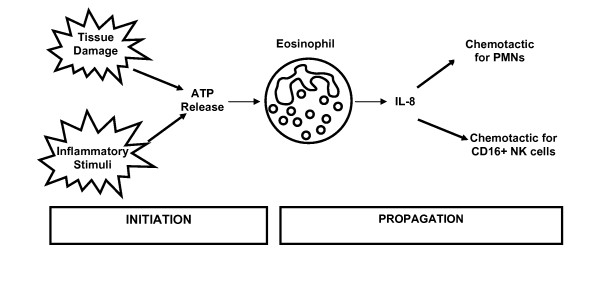
**Diagrammatic representation of the interplay between inflammatory mediators and cells**. Tissue damage or inflammatory stimuli results in ATP release which activates the P2X_7_R causing eosinophils to release IL-8 which amplifies the initial inflammatory response.

Ferrari *et al *[106] were the first to show that the P2X_7_R was present on eosinophils, with Mohanty *et al *[107] showing one year later that this expression was dependent upon stimulation by interferon-γ (IFN-γ). This stimulation-dependent expression contrasts with a more recent study which showed functional P2X_7_R were expressed endogenously on eosinophils and that inhibition of the P2X_7_R, abrogated agonist (BzATP) induced IL-8 release from eosinophils [108]. This is interesting in light of the observation that asthmatics secrete more IL-8 from their peripheral blood eosinophils than normal individuals [109]. Furthermore, as IL-8 is chemotatic for neutrophils [110] and CD16+ natural killer cells [111] this suggests a role for IL-8 in the initiation and propagation of the inflammatory response [108]. As ATP can be released upon tissue damage [104] and in response to inflammatory stimuli [49] (both of which may be present in asthma) it is possible that the P2X_7_R would be activated, resulting in IL-8 production and propagation of the immune response (Figure 3). This simplified description of part of the interplay between inflammatory cells and the mediators released, again suggests that the P2X_7_R may be a potential target for therapeutic intervention: however, these complex interactions are not yet fully understood. A better understanding of the basic pathophysiology of the initiation of inflammation will allow us to determine whether more specific therapies such as P2X_7_R regulation would prevent excessive inflammatory reactions, suppress acute inflammatory reactions and possibly augment the healing process following tissue damage [112].

## 4. Therapies directed at influencing the P2X_7_R

To date the majority of studies have focused on inhibiting the P2X_7_R to abrogate its downstream production of pro-inflammatory cytokines, with a number of reports now highlighting the potential benefit of P2X_7_R antagonists. Inhibiting the production of the undesirable excess of pro-inflammatory mediators such as IL-1β and TNF-α which cause the inflammatory state in many immune disorders is likely to be advantageous. In other circumstances, such as *M. tuberculosis *infection, activating the P2X_7_R may prove beneficial in bacilli eradication by encouraging infected macrophages to die by apoptosis rather than necrosis. This could introduce a number of problems, most notably being a systemic increase in inflammatory mediators and increased apoptosis in all cells expressing the P2X_7_R. A contrasting problem could exist for P2X_7_R antagonists, as the suppression of any natural P2X_7_R-dependent apoptosis could result in an increased susceptibility to autoimmune disease and carcinogenesis (*vide infra*). However, P2X_7_R-deficient mice have been described as having generally suppressed immune responses, without being immunocompromised [34]. Only when selective agonists and antagonists are widely available can any such assertions be addressed, although it is important to consider them as part of the broader recognition of the P2X_7_R as a potential therapeutic target.

### 4.1. The P2X_7_R, multinucleated giant cells and tuberculosis

In granulomatous disorders, monocytes or macrophages often fuse to form multinucleated giant cells (MGCs) [113], which results in increased cytokine production, non-phagocytic antigen internalisation, and disposal of infected or damaged monocytes. The antimicrobial activity of monocytes actually decreases with maturation to macrophages [114], whereas it is enhanced upon MGC formation [115]. An early study showed that the P2X_7_R may be important in the formation of MGCs [116], with Falzoni *et al *[117] speculating later that the P2X_7_R is involved in the final step of MGC formation (membrane fusion), as the receptor was found to cluster at sites of cell-to-cell interactions. They also showed that the P2X_7_R does not affect chemotaxis, cell aggregation or the expression of adhesion molecules and indicated that other factors may play an important role in the earlier stages of MGC formation [117]. However, there is new evidence to suggest that ICAM-1, in association with the P2X_7_R, may be important in this process [118-120].

Tuberculosis is a granulomatous disease caused by infection with *Mycobacterium tuberculosis *(*M. tuberculosis*), with the pathogen residing and replicating within macrophages. It still represents a major health burden, as a consequence of the emergence of antibiotic-resistant strains and co-infection with the human immunodeficiency virus (HIV) [121]. Following infection, part of the host immune response involves the initiation of a T-helper cell response against *M. tuberculosis*, with the subsequent activation of macrophages enabling them to become mycobactericidal [122,123]. This T-helper response also stimulates the formation of granulomas, which, as noted above, are characterised by P2X_7_R-expressing MGCs. In 1994 Molloy *et al *observed that apoptosis of an infected macrophage, but not necrosis, resulted in decreased mycobacterial viability [114] and that *M. Tuberculosis*-infected macrophages undergo apoptosis by a TNF-α-dependent mechanism [124,125]. However, pathogenic strains have been shown to reduce this TNF-α effect by increasing IL-10 production [126]. This anti-inflammatory cytokine then induces the release of soluble TNF-α receptor 2 (sTNFR2) from alveolar macrophages which inactivates TNF-α, thus inhibiting TNF-α-dependent apoptosis and ultimately favouring mycobacterial growth [126]. Interfering with this mechanism could therefore lead to the development of a new therapeutic strategy aimed at treating tuberculosis.

With P2X_7_R activation known to be associated with cell death, Lammas *et al *[127] suggested that the P2X_7_R may play a role in the apoptosis of infected macrophages and the accompanying mycobacterial death. The authors clearly showed that ATP-induced mycobacterial death was not a consequence of reactive oxygen or nitrogen species production, membrane disruption, or via any direct toxic effect [127]. The finding that apoptosis of infected macrophages is TNF-α dependent may provide an explanation as to why P2X_7_R are involved in mycobacterial death, however, to date, P2X_7_R-dependent TNF-α production has not been investigated in alveolar macrophages. Further evidence for involvement of the P2X_7_R in apoptosis of infected macrophages was provided in a study utilising P2X_7_R knockout mice [128]. However, again it was noted in this study that there are likely to be additional purinergic receptors that contribute to loss of mycobacterial viability, confirming an earlier observation by Sikora *et al *[129]. In 2000, it was found that extracellular ATP promoted the killing of virulent *M. Tuberculosis *in a phospholipase D (PLD) dependent manner [130], with further research suggesting that the mycobactericidal activity was due to *M. tuberculosis*-containing phagosomes fusing with lysosomes. ATP appeared to act through both P2X_7_R-dependent and independent mechanisms, with this process dependent upon increased cytosolic calcium and PLD [131]. More recently it has been shown that infection with the attenuated strain *M. tuberculosis *H37Ra inhibited P2X_7_R signalling [132] and in the same study cyclosporin A (an inhibitor of mitochondrial permeability transition (MPT), which is associated with increased mitochondrial cytochrome c release, necrotic macrophage death with resultant uncontrolled mycobacterial replication) was shown to re-establish P2X_7_R function in infected macrophages, and restore the antimycobacterial mechanisms associated with apoptosis [132].

Further evidence highlighting the potential importance of the P2X_7_R in tuberculosis has been provided by looking at receptor polymorphisms. Loss-of-function P2X_7_R polymorphisms have been shown to contribute to the variability in susceptibility to mycobacterial infections [133], perhaps through abolition of ATP-mediated killing of mycobacteria [134]. It appears that infected macrophages from individuals with polymorphisms in the P2X_7_R gene were resistant to apoptosis, which, as noted above, is important in the killing of intracellular mycobacteria [135,136]. It is therefore clear that the P2X_7_R should be investigated as a potential new therapy for treating tuberculosis.

### 4.2. The role of the P2X_7_R in cancer

The connection between inflammation and cancer was first described by Rudolf Virchow in 1863 (see reference [137] and references therein), with the interplay having been studied extensively since. For example, it has now been shown that there is an increased likelihood of a cancer developing at a site of chronic inflammation [138]. A polymorphism in the TNF-α promoter resulting in enhanced plasma TNF-α has been associated with an increased incidence of prostate cancer [139], while a polymorphism increasing IL-1β production conferred a greater susceptibility to gastric cancer [140,141]. Given the importance of the P2X_7_R in regulating cell death and cytokine production it is perhaps unsurprising it may play a role in cancer. Therefore, the development of either P2X_7_R agonists or antagonists may be useful anti-cancer agents, as agonists could kill cells, whereas antagonists would perhaps stop proliferation.

In 1996, T lymphocytes were found to express a purinergic receptor (suggested to be the P2X_7_R) which when inhibited, severely decreased cell proliferation [142]. Three years later these authors extended their findings by reporting that P2X_7_R transfection into lymphoid cells (lacking endogenous receptor expression), sustained their growth in serum-free medium [143]. They suggested that an ATP-based autocrine/paracrine loop existed which supported lymphoid cell proliferation in the absence of growth factors normally present in serum [143]. In isolation this was an important finding because one of the six alterations (the 'Hallmarks of cancer') thought to be essential in the transformation of a normal cell into a cancerous cell is 'self-sufficiency' in growth signals [144]. Recently it was shown that P2X_7_R transfection increased cellular energy stores (i.e. ATP) and the resting mitochondrial potential of transfected cells both of which gave the cells a growth advantage [145]. As mitochondrial dysfunction is important in apoptosis [146], any increase in resting mitochondrial potential would be expected to make cells resistant to apoptosis, thus providing them with a growth advantage [145] a further alteration thought to be essential in carcinogenesis – 'evasion of apoptosis' [144]. These observations are of clear importance given the earlier observation that the P2X_7_R is over expressed in several cancers [147].

In addition, the Glu^496^Ala P2X_7_R polymorphism discussed earlier produced a lack of agonist-mediated apoptosis in some patients with chronic lymphoblastic leukaemia [60]. In contrast, another report found that this polymorphism did not cause an increased risk of chronic lymphoblastic leukaemia [148], however the situation is clearly complex with different P2X_7_R polymorphisms found to contribute to the clinical outcome of chronic lymphoblastic leukaemia [149]. It is important that P2X_7_R polymorphisms and their associations with cancer be clarified, so that their potential as a prognostic tool can be determined. A new paper by Carta *et al *[150] has suggested that histone deacetylase (HDAC) inhibitors (novel agents currently being developed as pleiotropic anti-cancer agents) may have potential for development as anti-inflammatory agents as they reduced ATP-stimulated IL-1β production via the P2X_7_R. The potential role of P2X_7_R ligands in the treatment of cancer appears exciting and will undoubtedly be the subject of many future investigations.

## 5. Conclusion

In the 10 years since the purinergic P2X_7_R was cloned it is now clear that this receptor plays a number of important functions in the immune system. The importance of the P2X_7_R on macrophages is best understood, with the P2X_7_R playing an important role in the formation of MGCs and in macrophage intracellular killing of mycobacteria, such as *M. tuberculosis*. Moreover, the P2X_7_R is clearly involved in secretion of cytokines by macrophages (and other cells such as monocytes and microglia), particularly IL-1β, IL-18, TNF-α and IL-6, all of which play an important role in mediating inflammatory responses. The P2X_7_R has been shown to regulate the release of IL-8 from eosinophils and may be expressed on PMNs, potentially influencing their function. Although there is currently less evidence that the P2X_7_R regulates cytokine production in granulocytes, it appears to play a pivotal role in regulating apoptosis and cell death. Therefore, the P2X_7_R represents an exciting target for regulating peripheral and central inflammation and given the appropriate disease state, P2X_7_R antagonists may serve as a new class of anti-inflammatory compounds, capable of not only inhibiting the initiation of inflammation, but also potentially enhancing resolution.

## Abbreviations

ATP Adenosine 5'-triphosphate

BzATP 2', 3'-O-(benzoyl-4-benzoyl)-ATP

COX-2 Cyclooxygenase type 2

ICE Interleukin-converting enzyme

IL Interleukin

IL-1RA Interleukin 1 receptor antagonist

INF-γ Interferon-γ

LPS Lipopolysaccharide

MGC Multinucleated giant cell

oATP Oxidised adenosine 5'-triphosphate

P2X_7_R P2X_7 _receptor

PMN Polymorphonuclear neutrophil

TBI Traumatic brain injury

TNF-α Tumour necrosis factor-α

## Authors' contributions

MFL performed the literature review, wrote the first draft of the review and provided ideas and discussion related to the topic. DAS, DJD and JPH provided intellectual input and contributed to the writing of the review. AGR conceived the idea of writing a review, and along with JS and KF contributed to the structure and writing and provided significant editorial contributions to the content of the review.
